# Accelerating the Screening of Small Peptide Ligands by Combining Peptide-Protein Docking and Machine Learning

**DOI:** 10.3390/ijms241512144

**Published:** 2023-07-29

**Authors:** Josep-Ramon Codina, Marcello Mascini, Emre Dikici, Sapna K. Deo, Sylvia Daunert

**Affiliations:** 1 Department of Biochemistry and Molecular Biology, Miller School of Medicine, University of Miami, Miami, FL 33136, USA; jrc356@miami.edu (J.-R.C.); edikici@med.miami.edu (E.D.); sdeo@med.miami.edu (S.K.D.); 2 Department of Bioscience and Technology for Food, Agriculture and Environment, University of Teramo, 64100 Teramo, Italy; 3 Dr. John T. Macdonald Foundation Biomedical Nanotechnology Institute (BioNIUM), University of Miami, Miami, FL 33136, USA; 4 Clinical and Translational Science Institute (CTSI), University of Miami, Miami, FL 33136, USA

**Keywords:** machine learning, small peptides, molecular docking, classification algorithms

## Abstract

This research introduces a novel pipeline that couples machine learning (ML), and molecular docking for accelerating the process of small peptide ligand screening through the prediction of peptide-protein docking. Eight ML algorithms were analyzed for their potential. Notably, Light Gradient Boosting Machine (LightGBM), despite having comparable F1-score and accuracy to its counterparts, showcased superior computational efficiency. LightGBM was used to classify peptide-protein docking performance of the entire tetrapeptide library of 160,000 peptide ligands against four viral envelope proteins. The library was classified into two groups, ‘better performers’ and ‘worse performers’. By training the LightGBM algorithm on just 1% of the tetrapeptide library, we successfully classified the remaining 99%with an accuracy range of 0.81–0.85 and an F1-score between 0.58–0.67. Three different molecular docking software were used to prove that the process is not software dependent. With an adjustable probability threshold (from 0.5 to 0.95), the process could be accelerated by a factor of at least 10-fold and still get 90–95% concurrence with the method without ML. This study validates the efficiency of machine learning coupled to molecular docking in rapidly identifying top peptides without relying on high-performance computing power, making it an effective tool for screening potential bioactive compounds.

## 1. Introduction

Peptide-based therapeutics that use bioactive peptides have garnered increasing attention due to their remarkable versatility [1,2]. In 2021, peptide-containing drugs constituted 24% of all FDA-approved drugs, a significant increase from the 10% observed between 2016 and 2020, underscoring the growing interest in this field [3]. Various in vitro techniques, such as phage display, bacterial display, and yeast display, have been employed to identify lead candidates [4]. However, these methods demand considerable expertise and resources, and are costly and time-consuming, particularly when screening extensive libraries. Consequently, researchers often turn to in silico approaches for the initial design of peptide candidates, followed by in vitro and in vivo validation [5,6].

Protein-protein and peptide-protein interactions underpin most biological processes. As such, mimicking these interactions presents a powerful strategy for modulating mechanisms, enhancing, or inhibiting specific pathways, or detecting the presence of organisms in samples [7]. The inherent flexibility of peptides poses a challenge when calculating binding affinities for peptide libraries against proteins, often requiring substantial time investments [8]. 

Running molecular docking to large peptide libraries is a computationally expensive screening step used by many researchers [9,10,11]. It represents an important part of the investigation process, yet they can also be considered wasteful. This is because, despite the significant resources they require, only a small fraction of the highest-scoring compounds is typically selected for further experimental testing, highlighting the need for more efficient approaches in the field [12].

For this reason, researchers have increasingly turned to machine learning approaches to predict interactions, with the goal of accelerating the screening process [13,14,15,16,17]. In a recent review by Ye et al. [18] these methods have been categorized into five main groups: Linear-based, including linear regression and logistic regression; Tree-based, including decision tree, random forest, and gradient boosting machines; Kernel-based, including radial basis function, linear discriminate analysis, and support vector machine; Neural-network-based, including convolutional neural-networks, recurrent neural networks and generative adversarial networks; and Attention-mechanism-based, which includes transformers and BERT. 

Gradient boosting, first introduced in 1999 by Friedman [19], has been significantly developed by the community through open-source packages in Python and R programming languages, like the package ‘lightgbm’, used in this paper [20]. It is a particularly advantageous choice for predicting peptide-protein interactions as it can handle complex non-linear relationships, perform automatic feature selection, and provide interpretable models, making it well-suited for biological data [21]. Among these, LightGBM has gained traction for its rapid processing. Although previous studies employing sequence-based, tree-based methods for peptide-protein interactions predictions primarily utilize a Random Forest (RF) framework [16,17,22,23], our approach aims to predict binding for hundreds of thousands of peptides, necessitating speed as a crucial factor. Therefore, we think LightGBM can be more suitable to achieve our goal than RF.

In this work, we present a novel approach that combines rapid molecular docking and sequence-based prediction using the LightGBM framework. Our goal is to accelerate the peptide screening process by predicting which peptides will produce better docking results and guide the researchers to a more targeted docking. 

To validate the pipeline, we use binding score data from molecular docking experiments carried out using as ligands the entire tetrapeptide library, comprised of 160,000 peptides. The target binding sites selected were the glycosylation site from the envelope protein of four different viruses: Chikungunya (CHIKV), Dengue (DENV), West Nile (WNV), and Zika (ZIKV). All of these are viral diseases transmitted by mosquitoes, and they belong to the *Flaviviridae* family (except for the Chikungunya virus, which belongs to the *Togaviridae* family). The molecular docking was performed using Openeye software [24]; additionally, the same receptors were also used to perform docking with AutoDockFR [25] to prove that different software does not change the performance of the model. Both tools were used in a rigid setup, through an ensemble docking method. These methods sample peptide conformations as a pre-processing step without any prior knowledge of the receptor. These conformations are then docked rigidly or semi-rigidly into the receptors, a technique that has been studied and documented to yield good accuracy for small and medium-sized peptides, typically less than or equal to 9 amino acids, like the tetrapeptides studied here [26,27,28]. However, it is wort noting that the robustness of the models does not primarily depend on the specific docking software used. The models are designed to predict the best peptides based on the distribution provided by the docking software they are trained with. Therefore, the key to a successful model lies not in the software chosen for docking but in the quality and distribution of the docking results utilized during the model’s training phase. Finally, a case study was performed using a different molecular docking software, AutoDock CrankPep (ADCP) [29], which was specifically developed to study peptide-protein interactions by considering peptide flexibility. The utilization of ADCP provides further evidence supporting the software-agnostic nature of our research methodology.

The 3-D protein structures listed in the Protein Data Bank, with accession numbers 3N40 for CHIKV, 4UTC for DENV, 3I50 for WNV, and 5IRE for ZIKV were used for the docking [30,31,32,33]. 

**Figure 1:**
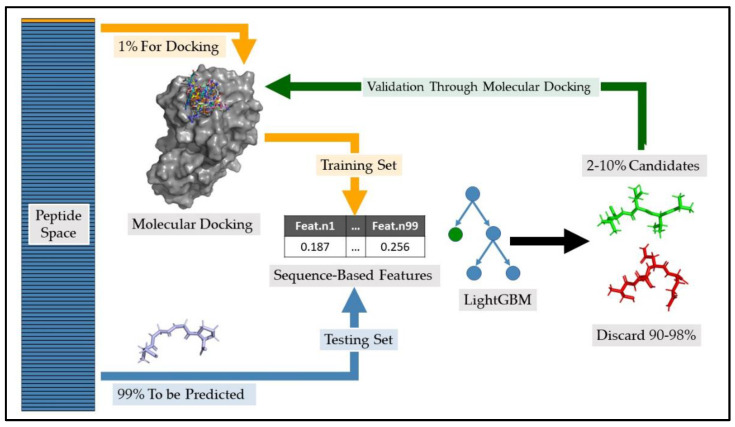
Overview of the process for training and testing the LightGBM in binary classification model. Initially, a random 1% subset of the tetrapeptide space undergoes molecular docking. The docking results are then categorized into better 20% performers and worse 80% performers. This category is the target of the classification task. Ninety-nine sequence-based features from each peptide were extracted and incorporated into a matrix alongside the target variable, forming the training data for the LightGBM algorithm. The remaining 99% of the tetrapeptide space, which does not undergo docking, is similarly processed to obtain sequence-based features which serve as the testing data for the algorithm. A subset of 2–10% of peptides having the highest probability of being better performers are selected. The selected peptides undergo a second molecular docking to validate the goodness of the selection process.

While these specific datasets were used in this research, our approach is designed to be a versatile tool applicable to all proteins with a 3-D structure, spanning all species and biological contexts. An overview of the process of the proposed method is represented in Figure 1.

## 2. Results and Discussion

### 2.1. Molecular Docking Results

**Figure 2:**
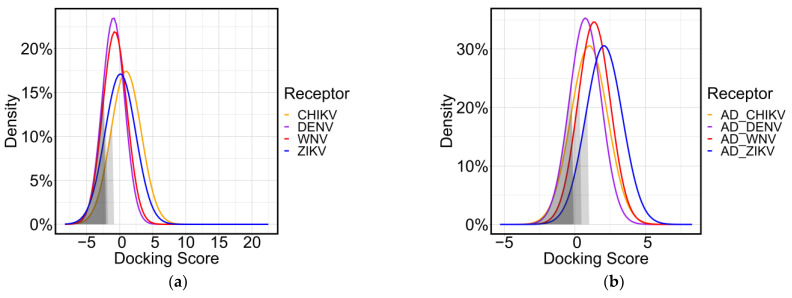
**Figure 2**. Gaussian distribution of the docking scores obtained by molecular docking all tetrapeptide library to the four envelope protein targets using Openeye software (**a**), or AutoDockFR software (**b**).

The binding score of the entire tetrapeptide library, taken as ligands, was calculated against the four receptors represented by the four different envelope proteins glycosylation binding site (CHIKV, DENV, WNV, and ZIKV). The 3-D protein structures were downloaded from the Protein Data Bank, with accession numbers 3N40 for CHIKV, 4UTC for DENV, 3I50 for WNV, and 5IRE for ZIKV. The binding score was calculated using two different docking software, Openeye and AutodockFR suite, to have two different molecular docking approaches to confirm that the ML procedure was not software dependent [24,25]. The results of the binding score calculated by the two molecular docking software were reported in Figure 2 for Openeye (a) and for AutodockFR suite (b) in the form of gaussian distribution highlighting the two groups selected for the ML classification (20% better performers and 80% worse performers). All docking runs had a normal behavior with slight differences in the average and standard deviation of the gaussian curve.

The score values calculated by the molecular docking function were used to estimate the affinity of peptides for proteins. Lower values indicate a more favorable docking outcome between the peptide and the protein. This is because lower values represent a more favorable interaction between the two molecules. 

The overall binding tendency showed a similar trend between binding sites. This is justified by the molecular docking functions that use noncovalent interactions, particularly hydrogen bonds, to screen compounds that could potentially interact with the binding site.

The results of the molecular docking study were likely to be representative of the diversity of peptides that could bind to a binding site. In fact, the peptide library ligands consisted of all the tetrapeptide space, 160,000 peptides. Moreover, although the four target proteins are from the same viral family, it’s important to note that the glycosylation sites within these proteins showed the greatest variation in amino acid composition, hence creating distinct chemical environments. This ensured that the results were as variable as possible, as each peptide in the library had a different amino acid sequence and the glycosylation binding sites had a high degree of amino acid sequence variability.

ADCP results are discussed in Section 2.5 Case Study.

### 2.2. Machine Learning Algorithm Selection

A comparison between various ML methods in terms of processing time, F1-score, and accuracy, was done using the WNV molecular docking dataset, (Table 1). Notably, all methods demonstrated similar F1-scores and accuracy ranging from 0.52 to 0.56 and from 0.77 to 0.86 when using a probability threshold of 0.5. However, a distinct disparity was observed in computation times. The LightGBM algorithm outperformed all others with a significantly lowest processing time of 0.057 min. For comparison, the next fastest algorithm, Naive Bayes, required 0.874 min. The most time-consuming methods were Random Forest (RF) and SVM, which took 326 min and 1690 min, respectively, to finalize the processing. Given similar F1-scores and accuracy across methods, it becomes crucial to consider computation time as a deciding factor. Therefore, considering the balance of accuracy and computational efficiency, the LightGBM method appeared to be the optimal choice for the tetrapeptide dataset.

### 2.3. Evaluation of LightGBM Models

One of the primary considerations for our model task centers around determining the size of the group of peptides that yield more favorable docking results while maintaining a high level of performance and a small set of training peptides to ensure the efficacy of the method. In that regard, our initial step was to fine-tune the model’s hyperparameters for different group sizes.

#### 2.3.1. Group Selection and Hyperparameter Tuning

Hyperparameter optimization is crucial in machine learning, as it can significantly impact model performance. Identifying the optimal set of hyperparameters enhances a model’s generalization ability, leading to more precise predictions and improved outcomes. Our objective is to pinpoint the smallest groups of ‘better performing’ peptides and the smallest training peptide groups, while maintaining a good model performance. Consequently, we performed a fast Bayesian hyperparameter search, with 15 initial points and 15 iterations, across a range of groups to identify the best results. 

Combinations of different training peptide groups (1% to 10%) and better-performing groups (1% to 40%, plus a 67% value, representing the difference between positive and negative docking scores) were tested. The metric utilized to optimize the performance of the model was F1-score, which is a harmonic balance between precision and sensitivity suitable for imbalanced datasets like this one, which has a small group (“better performers”) and a large group (“worse performers”). 

Our findings, shown in Table 2, indicate that there is a strong correlation between the F1-score and the size of the better-performing group. When the size of the better-performing group was 20% or higher, the F1-score was over 0.5, indicating a satisfactory level of accuracy. On the other hand, we don’t see a correlation between F1-score and the size of the training set. Detailed results are provided in the Supplementary Materials (Table S1, Figure S1). 

Based on these findings, setting the better-performing group to 20% and the training group to 1%, was a good compromise yielding an F1-score close to 0.6. This means that we can do an initial docking with 1% of the tetrapeptide library (1600 peptides) and train the LightGBM algorithm to predict which peptides from the whole tetrapeptide space (160,000 peptides) would be the 20% better performers. 

With these group sizes, a deeper Bayesian hyperparameter optimization, with 25 initial points and 15 iterations, was performed using the same search space, and to further enhance the robustness of our results, this search was repeated 50 times to determine the final best hyperparameters. The selected hyperparameters were: num_leaves: 29; max_depth: 2; learning_rate: 0.014; scale_pos_weight: 3; min_data_in_leaf: 35; feature_fraction: 1; bagging_freq: 5; pos_bagging_fraction: 0.71; neg_bagging_fraciton: 0.84. A deeper explanation about these parameters and the search space used can be found in the Section 3.

#### 2.3.2. Performance of the Models 

The performance of each model was evaluated using five different metrics, namely the Area Under the Curve (AUC) of the Receiver Operating Characteristic (ROC), accuracy, specificity, sensitivity, and F1-score.

**Figure 3:**
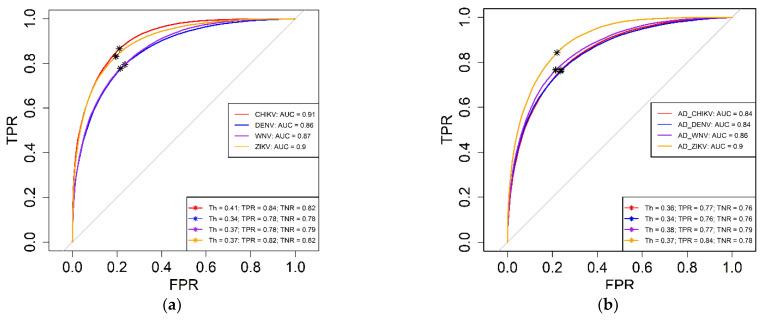
Performance of the prediction for different datasets. AUC-ROC values are used to compare the performance of different datasets. The ROC curve was extracted three times after three different random subsampling, obtaining similar results. Legend shows the average AUC of the triplicates, and the best threshold for each dataset. (**a**) Openeye datasets (**b**) AutoDockFR (AD) datasets. AUC: Area Under the Curve; Th: Threshold; TPR: True Positive Rate; TNR: True Negative Rate; AD: AutoDockFR.

The hyperparameters obtained in the previous section were employed to evaluate all datasets. The AUC-ROC was calculated in triplicates for all datasets. The triplicates forecast very similar results in each run. The triplicates were averaged, and the values obtained were plotted in Figure 3. AUC-ROC values vary between 0.84 and 0.91 across the datasets.

AUC-ROC is a useful metric that provides a single value reflecting both the true positive rate (TPR) and false positive rate (FPR) of a model. This enables a robust comparison of classification accuracy across various models. Our AUC-ROC values suggest that our model performs reasonably well across the datasets evaluated. Furthermore, with the ROC curve, we can calculate the best classification threshold to increase the sensitivity (TPR) and specificity.

To further evaluate the models, a Monte Carlo cross-validation approach was implemented, which involved 100 random subsamples for each dataset, then the metrics obtained were averaged. For each iteration, the dataset was randomly split into two subsets: 1% for training the algorithm and 99% for testing. The results shown in Table 3, indicate that we can obtain a prediction with an accuracy oscillating between 0.83 and 0.85, an F1-score between 0.6 and 0.67, a sensitivity between 0.64 and 0.76, and a specificity between 0.86 and 0.88. Values for each Monte Carlo iteration are available in Table S2.

Additionally, to prove that our approach is not software-dependent, the AutoDockFR datasets were also evaluated. The results, in the second part of Table 3, were like those obtained with the Openeye datasets, with an accuracy ranging from 0.81 to 0.84, an F1-score between 0.58 and 0.65, a sensitivity between 0.64 and 0.72 and a specificity between 0.86 and 0.87.

The prediction threshold for a classification task, which is the cut-off value that determines the class label for a given prediction probability, was also examined in our study. In the datasets studied, we observed that sensitivity increases within a threshold range of 0.33 to 0.41. This behavior can be attributed to the imbalanced nature of the data. The datasets are unequally divided, with one group representing 20% of the total data (“better performers” group) and the other, a significantly larger portion, representing 80% (“worse performers” group). 

**Figure 4:**
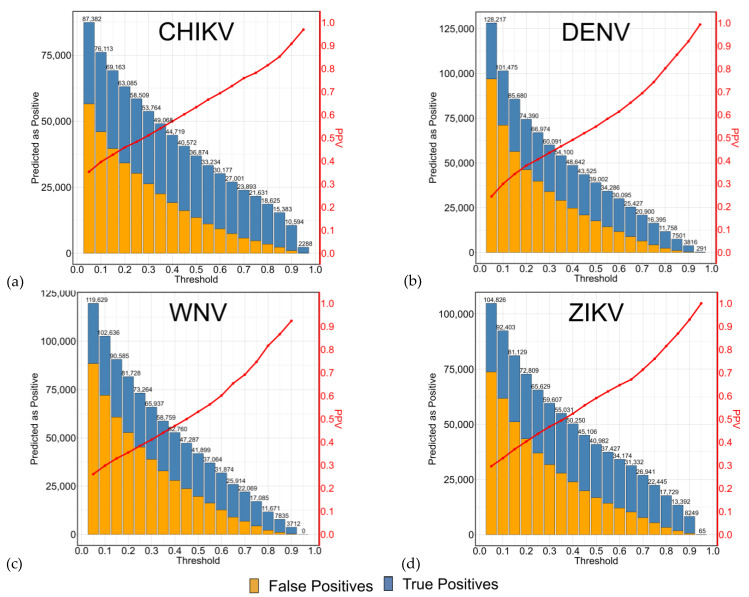
Positive Predictive Value, True Positives and False Positives representation for the Openeye datasets. Positive predictive value (PPV), in red, represents the probability that an observation with a positive predicted outcome is indeed positive. It is obtained by dividing the true positive values by all positively predicted values. The plots (**a**–**d**) show the PPV (red), true positives (blue) and false positives (orange) across different prediction thresholds for (**a**) CHIKV, (**b**) DENV, (**c**) WNV, (**d**) ZIKV datasets.

Upon lowering the prediction threshold, we inevitably include more false positives in the data. However, given the size discrepancy between the negative and positive groups, we still maintain a substantial count of true negatives. This sustains high specificity while concurrently improving sensitivity. If the prediction threshold is lowered, approximately 50,000 peptides would be predicted as positive, which constitutes nearly one-third of the entire tetrapeptide space. Moreover, with a threshold approximating 0.35, the positive predictive value (PPV) hovers around 50%, as shown in Figure 4. Figure 4 shows Openeye datasets; AutoDockFR datasets (Figure S2) present similar results.

The process of selecting an appropriate prediction threshold is critical and must be delicately handled in accordance with specific circumstances. Opting for a higher prediction threshold can lead to a manageable number of candidate peptides (true positives), at the risk of overlooking certain potential candidates. On the other hand, a lower prediction threshold could ensure the selection of all possible candidates but may also include some false candidates (false positives) in the process. 

While the observed increase in sensitivity, accompanied by a robust specificity, is of notable interest, it diverges from the primary objective of this research. Our central aim is to identify peptide candidates and reduce the time expended to evaluate the whole peptide space. With a classification threshold of ~0.35, out of the 50,000 peptides predicted as positive, only half are true candidates, while the remaining half are false positives. This already constitutes a significant time reduction when docking one-third of the peptides as opposed to the full library. However, with larger peptide spaces in mind, such as pentapeptide (3.2 million peptides) or hexapeptide (64 million peptides), we strive for even greater efficiency.

The key to verifying the authenticity of selected candidates lies in a subsequent step of peptide docking. The predictive model’s threshold, therefore, should be carefully calibrated based on the computational resources and time available to the research team. A high threshold may be suitable if computational power is limited, ensuring that only the most likely candidates are selected. In contrast, a lower threshold would be more computationally demanding but would ensure no potential candidates are missed. This subject is further discussed in the Section 2.4 LightGBM versus Molecular Docking Results where we compare this method to a molecular docking method without ML. 

The classification task yielded promising results, however we decided to evaluate the application of the LightGBM algorithm for a regression task, with the goal of predicting precise docking scores. Like before, the model was trained with 1% of the dataset, and tried to predict the remaining 99%. As in classification, the training matrix consisted of the docking score plus 99 features that were extracted for each peptide as described in the Section 3. The overall performance of this regression model yielded outcomes that were measured by Root Mean Square Error (RMSE), using as reference the molecular docking score obtained by Openeye software. The regression prediction (Figure S3) revealed notable differences in the RMSE values across different ranges of the molecular docking score distribution. The RMSE for the entire dataset was 1.2. However, when the dataset was divided into three subsets—lower bound (worst performing peptides), middle bound, and upper bound (better performing peptides)—distinct variations in RMSE values were observed. The lower bound and upper bound subsets, representing the tails of the score distribution, showed significantly higher RMSE values of 1.9 and 2.3, respectively. These results indicate that the model’s prediction accuracy was notably poorer for scores at the extremes of the distribution, which are the ones we are most interested in, specifically the top peptide ligands.

In contrast, the RMSE for the middle-bound subset, representing the bulk of the data, was only 1.04. This suggests that the model performed relatively well in predicting scores in this middle range but struggled when predicting more extreme scores.

These results highlight a key limitation of the regression-based approach: its diminished predictive accuracy for scores at the tails of the distribution. This means that we cannot successfully predict a molecular docking score using the regression approach. As such, we continued with the classification approach.

#### 2.3.3. Importance of Choosing the Right Features

Choosing the right features, i.e., peptide properties that the model uses to make accurate predictions, is important because they provide the necessary information for the LightGBM algorithm to learn and discern patterns. These peptide properties can significantly enhance the predictive power of the model as they enable the model to capture the underlying relationships that determine the outcome. Furthermore, the interaction between these features can reveal complex dependencies that a simpler model might miss. The features used in this paper are obtained from the ‘Peptides’ R package (https://github.com/dosorio/Peptides/ (accessed on 12 June 2023) [34]). All sequence-based properties from this package were considered. However, following a comprehensive screening of similarities across the entire tetrapeptide library via Principal Component Analysis (PCA), only 99 properties were chosen from the potential 168 properties. More detailed information about these properties can be found in the Section 3. 

By analyzing the importance of each feature post-training, we can gain valuable insights into which peptide properties are most influential in our predictions, guiding future research and model refinement. The five most important features for all datasets are reported in Table 4. 

In this table, it becomes clear that even with very similar datasets, the same algorithm, and an identical feature set, each dataset seems to highlight different essential features. This can be attributed to several reasons. 

First, each dataset, while similar, is not identical. Subtle differences in peptide composition or distribution can lead to variations in the impact of individual features. For example, if one dataset is representing a small binding pocket, the molecular weight will most likely be a very important feature, while if it represents a big binding pocket the size will not be as limiting, and the molecular weight will be less important. 

Second, different software tools use different scoring functions to predict binding affinity, meaning they emphasize different features. These differences in emphasis can, in turn, affect the importance of certain features when training machine learning models on these data. Many molecular docking software tools consider steric (van der Waals) interactions, hydrogen bonding, electrostatic interactions, solvation effects, and entropy effects in their scoring functions. However, they may weigh these parameters differently, leading to different importance scores in feature analysis post-training. The importance of all features that were used in this study can be found in the Supplementary Figure S4 and Table S3. 

### 2.4. LightGBM Versus Molecular Docking Results

To compare the results obtained from our new method, which incorporates molecular docking and machine learning, we referred to our previously utilized approach as a benchmark [11,35]. This conventional method involved a two-step process: first, molecular docking of the entire tetrapeptide library, consisting of 160,000 peptides, was performed, followed by a comprehensive structural analysis. This analysis compares amino acid distribution at each residue position (i.e., first, second, third, or fourth) between the top and bottom 5% of performing tetrapeptides. Analyzing residue occurrences that show significant divergence between these two groups, at the 90% confidence level, generates a selection of tetrapeptides, which are then trimmed to include only those that fall within the top 5% of docking scores.

The new strategy introduced here consists of docking 1% of the tetrapeptide space (1600) and classifying the remaining 99% of the library as better performers and worse performers using ML algorithm, LightGBM. Then a classification threshold is selected to obtain a reduced selection of peptides. Finally, this selection is validated by docking the selected peptides to the target to obtain a binding score. As mentioned earlier, the selection of the prediction threshold is a critical step that establishes a balance between the time taken for the second docking step and the accuracy in detecting true candidates. By carefully adjusting this threshold, we can optimize the trade-off between computational efficiency and the precision of candidate identification. 

To select the best threshold we consider two factors, the first one is the number of peptides classified as better performers with that threshold. Since we will need to dock these peptides, we want to choose a small group that, at the same time, includes as many real better performers as possible. This number of selected peptides will determine the size of the second docking where we will check if the candidates selected are indeed ranked as better performers. The second factor we consider is the comparison of peptides selected through our proposed method with those selected using the former method of docking and structural analysis without ML. We calculate the concurrence as a percentage, which is determined by dividing the number of peptides selected by the proposed method that are also selected by the conventional method divided by the number of peptides selected by the conventional method only. This gives us a measure of how well the proposed method aligns with the conventional approach. Results are represented in Table 5 as an average of all datasets for each molecular docking software. 

The results obtained provide strong evidence in support of our newly proposed approach, which combines rapid molecular docking with subsequent machine learning. This methodology was found to be highly effective and on par with the conventional method in the identification of peptide candidates. The high concurrence rates substantiate the efficacy of our proposed strategy.

The choice to increase the prediction threshold, and therefore reduce the number of peptides to dock, should be taken carefully and knowing that the lower the number of peptides to dock the higher the risk of overlooking some candidates. Nevertheless, we can see in Table 5 that by choosing the most probable 16,000 better binders, we are reducing the time by a factor of 10 while keeping 90–95% of the peptides that would have been selected by the conventional method. By selecting the most probable 8000 peptides we would be getting between 81–85% concurrence with the conventional method while reducing the time by a factor of 20. In that regard, our proposed method demonstrates a remarkable combination of accuracy and efficiency. Future work could explore methods to further optimize this balance.

It is important to note that the pool of 8000–16,000 peptides represent the maximum number to be molecularly docked and it can be further refined based on the specific objectives of the researcher. If a researcher is particularly interested in certain functional groups or peptide properties, such as levels of hydrophobicity or the presence of positive or negative charges, they can filter the peptides accordingly, since these are theoretical properties. This refinement should be performed prior to the second round of docking, as it can significantly reduce the computational time required for the screening process.

### 2.5. Case Study

Here, we provide a practical example of our methodology, applying each step in sequence. Initially, we randomly selected 1600 tetrapeptides, i.e., 1% of the sample pool. These are subjected to a flexible docking procedure against the WNV receptor using AutoDock CrankPep (ADCP) a tool featured in ADFR software specifically designed for peptide-protein docking [29]. This tool is capable of automatically generating 3-D structures of peptides. However, as it only accepts peptides containing five or more residues, we appended a Gly residue to the end of all tetrapeptides in the sample.

Upon the completion of docking, we extracted features for both the random 1% sample and the remaining 99% of the peptide library. Subsequently, we divided the random 1% sample into two groups to establish the target labels for the LightGBM classification task: “better performers” and “worse performers”. The algorithm training was carried out using the random 1% sample and the hyperparameters obtained in Section 2.3.1—‘Group Selection and Hyperparameter Tuning’. The trained model was then deployed to predict the remaining 99% of the library.

Following the prediction, we ranked the full list of peptides according to their predicted probability of falling into the “better performers” category. It’s important to remember that the selection of the probability threshold, which determines the number of peptides chosen for the second docking round, is dependent on the available resources of the researchers. In this case study, we selected the top 8000 peptides, equivalent to a probability threshold of 0.83. For illustration purposes, we also included the bottom 8000 peptides, those with the least likelihood of being categorized as “better performers”, even though this would not typically be done in a real-world application of the method.

**Figure 5:**
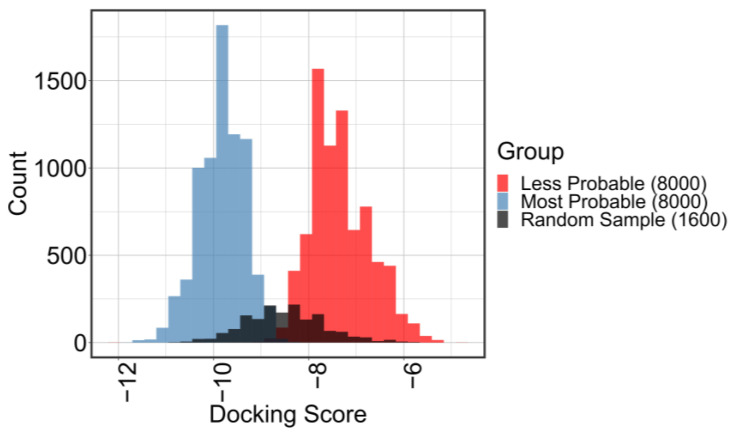
After using the results from the random sample of 1% of the library (gray) to train the LightGBM algorithm, the remaining 99% is predicted. The peptides are ordered based on their probability to belong to the better performers group, and 8000 peptides with the highest probability are chosen for docking (blue). To better illustrate the case study, the 8000 peptides with the lowest predicted probability to belong to the better performers group (red) is also docked.

A second round of flexible docking was conducted for these groups, using identical parameters as the first round. The docking results for these three groups (random 1% sample, top 8000, and bottom 8000) are presented in Figure 5, which illustrates a significant difference (mean top: −9.8; mean bottom: −7.3; *p* < 0.005; CI95: 2.44–2.48) between the top and bottom groups and displays a normal distribution for the random sample.

As shown in Figure 5 the model built using the 1% random sample worked very well in predicting the tails behavior of the gaussian distribution. Considering 1% sample as representative of the tetrapeptide space, the peptides that have a docking score below the docking score of the best 20% of this sample (score = −9.2) are in the “better-performers” group, while the peptides with a docking score above this are in the “worse-performers” group. The validation step confirmed that 87.4% of the 8000 peptides with the highest probability to belong to the “better-performers” group are in that group. On the other hand, 99.9% of the 8000 peptides with the lowest probability to belong to the “better-performers” group were correctly classified as “worse-performers”. These preliminary results confirmed the success of the pipeline proposed by this work.

In order to maximize the utilization of resources, we conducted the flexible docking across all available nodes of the Pegasus Supercomputer cluster of the University of Miami. The computational capacity of these nodes varied; some had 8 cores, while others had 16 cores available. ADCP is able to detect the available cores and distribute the docking calculations accordingly in parallel. When performed with 8 cores, each peptide docking process lasted approximately 29 min. However, with 16 cores, the docking time was reduced to about 7 min. Here we used all available nodes, consisting of a mix of 8 and 16 cores nodes. We calculated the average docking time per peptide which was found to be 15.33 min. It’s worth noting that the number of available nodes in the cluster varies depending on current demand. The task of docking 17,600 peptides, fully utilizing the capacity of the University’s supercomputer, took us approximately 10 days. By extrapolation, considering we used the full supercomputer capacity, we can estimate that docking an additional 90% peptides would have required roughly 90% more time, translating to a duration exceeding three months. 

Docking the top 8000 peptides would likely have taken under 5 days, considering the increased availability of resources that would result from the bottom 8000 peptides not occupying those resources. This underscores the substantial difference between conducting a broad-spectrum peptide docking process that would exceed three months, versus performing a targeted docking approach facilitated by machine learning, which would take a maximum of 5 days. This not only optimizes resource allocation but also drastically reduces the time needed for the procedure, underscoring the efficacy and scientific value of this machine learning-guided approach. It is worth noting that the specific time taken will depend on the researcher’s resources and computational power, and that this example is used only to illustrate our point.

All the files to replicate the case study are in Supplementary Materials. Table S4: random sample docking results; Table S5: prediction results for 99% remaining; Table S6: docking scores for top 8000 peptides; Table S7: docking scores for bottom 8000 peptides; Scripts are in the scripts folder. 

## 3. Materials and Methods

### 3.1. Molecular Docking

Molecular docking calculations were performed using a high-performance computer equipped with 19 Intel Xeon X5690 processors running at 3.47 GHz, and 94.5 GiB of RAM. The system was operating under the Kernel Linux 2.6.32–642.1.1el6.×86_64, GNOME 2.28.2 environment. Molecular docking results using Openeye were obtained as described by Mascini et al. [11]. In the first step peptide libraries were designed and prepared using HyperChem 8.0.5 [36]. Peptides libraries were designed in zwitterionic mode using the Amber molecular mechanics method. Hydrogen atoms were added at pH 7, and the “Steepest Descent” algorithm was used to achieve convergence at 0.08 kJ mol^−1^ in 32,767 cycles. The secondary structure setting was set to default (beta-sheet). The script running in HyperChem automatically eliminated peptide duplicates. In the second step each peptide library was compacted into a single file and fast minimized in a vacuum using Openeye software tools. The energy minimization process was conducted using SZYBKI 1.5.7 in its default parameterization [37]. Ten conformers were generated for each peptide using OMEGA 2.4.6 with MMFF as the force field [38]. 

In the third step, the active grid box along with the multi-conformer rigid body docking were carried out using OEDocking 3.0.0 [24]. The envelope proteins used as receptors were from four different targets: Chikungunya (CHIKV), Dengue (DENV), West Nile (WNV), and Zika (ZIKV) viruses. The 3D protein structures are listed in the Protein Data Bank, with accession numbers 3N40 for CHIKV, 4UTC for DENV, 3I50 for WNV, and 5IRE for ZIKV [30,31,32,33].

ADFR suite was also used to prove the cross-software ability of our model. The peptide library was also prepared differently from before using a Python script to automate the molecule building through PyMOL (Schrödinger). The script build_proteins.py can be found in the Supplementary Materials. Initially, protein sequences are read from a file and then processed in parallel threads according to the number of available CPU cores. Each sequence is turned into a peptide structure through a process of building, geometry optimization, and hydrogen removal. Each optimized structure is saved as a pdb file in the designated output directory. This method allows the efficient creation and saving of peptide structures derived from an extensive list of sequences. This library was prepared using a laptop with an Intel i7-11800H processor with 64 GiB of RAM. Once the molecules are built, all remaining hydrogens are removed and then added using the tool ‘reduce’ with default parameters. Receptors were prepared using the tool ‘prepare_receptor’ and ligands were prepared using the tool ‘prepare_ligand’, both with default configuration. These tools transform the pdb-formatted files into AutoDock’s format pdbqt. The target file was generated using the tool ‘agfr’. The grid box was built manually using the same residues used for building the grid box for Openeye procedure. The binding pockets were determined using AutoSite1.0, and all binding pockets identified were selected. The target files were saved with trg format. The molecular docking was run in the same cluster as the OpenEye docking. The tool ‘adf’r was used with the parameters --nbRuns 2 --maxEvals 10000 -T -NG backbone. Results were processed by extracting the best result from each run. ADFR suite was also used in the case study as described below.

### 3.2. Case Study

The case study was performed using the ADFR suite with flexible docking with the tool ‘adcp’ (AutoDock CrankPep) [29]. This tool is specifically designed to evaluate peptide-protein interactions. First, a random sample of 1600 (1%) tetrapeptides was selected. The same tool generates the library; however, it only works with pentapeptides or larger peptides, therefore, a Gly was added at the end of all the tetrapeptides tested. The parameters used were, -t H3i50_E.trg -s SEQUENCE_HERE -c 8 -N 50 -n 5000000. 1 million steps per amino acid was chosen as recommended by the developers (5 million total -n) and 50 replicas. After the docking was performed, the LightGBM algorithm was trained with the results using the best parameters obtained in Section 2.3.1 Group Selection and Hyperparameter Tuning, and the features described in next Section 3.3 Datasets and Feature extraction. Once trained, the algorithm was then utilized to predict the performance group for the remaining 99% of the peptides in the library. Finally, the peptides predicted as top 5% (8000 peptides) were chosen as well as the bottom 5% (8000 peptides) to compare. The *adcp* tool was then used again with the same parameters to obtain a docking score. To compare both groups, after checking the equality of variance and normality, a Student’s *t*-test was performed.

### 3.3. Datasets and Feature Extraction

The datasets utilized in this study were obtained as described above. Four different targets from: CHIKV, DENV, WNV, and ZIKV capsid proteins were evaluated. Each dataset contained results from 160,000 tetrapeptides docked. 99 numerical sequence-based features were extracted using the R package ‘Peptides’ (https://github.com/dosorio/Peptides/ (accessed on 12 June 2023) [34]) and a binary target variable, 1 or 0, which represents the ‘better performers’ group and the ‘worse performers’ group respectively. 

All features from the ‘Peptides’ package can be deeply explored using the package reference manual (https://cran.r-project.org/web/packages/Peptides/Peptides.pdf (accessed on 12 June 2023) ). Here, we selected the following features from the package: Cruciani properties (physicochemical properties of peptides calculated using Cruciani’s method, which includes molecular weight, molecular volume, and other properties) [39]; amino acid indices used to describe the physicochemical properties of the amino acids present in the peptide (FASGAI vectors [40], Kidera factors [41], ProtFP [42], T-scales [43], VHSE scales [44], Z-scales [45]); isoelectric point (pI) computed using different pK scales (EMBOSS, Bjellqvist, Lehninger, Murray, Rodwell, and Sillero) [46]; mass shift for 15N (the mass shift of the peptide when all nitrogen atoms are replaced by 15N isotope); charge (the net charge of the peptide at pH 7) [47]; hydrophobicity, calculated using various hydrophobicity scales (Aboderin, AbrahamLeo, BlackMould, BullBreese, Casari, Chothia, Cid, Cowan3.4, Cowan7.5, Eisenberg, Fasman, Fauchere, Goldsack, Guy, HoppWoods, interfaceScale_pH2, interfaceScale_pH8, Janin, Jones, Juretic, Kuhn, KyteDoolittle, Levitt, Manavalan, Miyazawa, octanolScale_pH2, oiScale_pH2, oiScale_pH8, Parker, Ponnuswamy, Prabhakaran, Rao, Rose, Roseman, Sweet, Tanford, Welling, Wilson, Wolfenden, Zimmerman) [48,49]; amino acid index (aIndex, a measure of the relative volume occupied by the side chains of the amino acids in the peptide) [50]; Boman index (estimate of the peptide’s binding potential to other proteins) [51]; helical moment (hmoment, a measure of the amphipathicity of the peptide, calculated for two different angles, 100 and 160 degrees) [52]; instability index (instaIndex, a measure of the peptide’s stability in a test tube) [53]; and molecular weight (mw1, the monoisotopic molecular weight of the peptide) [49]. The importance of these features varies depending on the dataset (Table S3, Figure S4). The treatment of the datasets can be summarized as follows:

1. The datasets containing the tetrapeptide sequences and the molecular docking scores were combined with the peptide’s properties.
2. A binary target variable (0 or 1) was added to distinguish between ‘better performers’ and ‘worse performers’ groups. The size of these groups varied depending on the stage of the process. A range between 1% to 40% for ‘better performers’ and 60% to 99% for ‘worse performers’ groups was evaluated.
3. The datasets are divided into train and test sets. Train sets varying from 1% to 10% were evaluated.

### 3.4. Algorithm Selection

Other algorithms were tested using the dataset from WNV dataset. 1600 peptides were selected as train and 158,400 peptides were selected as test set. The R package ‘caret’ was used to evaluate different models available in the package, namely: Naive Bayes, Recursive Partitioning and Regression Trees (RPART), Gradient Boosting Machine (GBM), Neural Network (NNET), K-Nearest Neighbors (KNN), Random Forest (RF), and Support Vector Machine (SVM). The Light Gradient Boosting Machine framework is not available in ‘caret’ package, so the ‘lightgbm’ package was used. 

### 3.5. Light Gradient Boosting Machine

A binary classification task was performed using Light Gradient Boost Machine (LightGBM) where we wanted to distinguish between better performers and worse performers. We selected LightGBM for our analysis based on its speed advantage over other tree-based methods and machine learning frameworks. Moreover, LightGBM presents suitable features like, scalability, it is specifically designed for large datasets, making it an ideal choice for handling the 160,000 peptides in our study; computational efficiency, reduces memory usage and training time by employing a histogram-based algorithm; and robustness to overfitting, thanks to gradient-based one-side sampling (GOSS) and exclusive feature bundling (EFB). More information about the LightGBM algorithm can be found in the documentation page (https://lightgbm.readthedocs.io/en/v3.3.2/index.html (accessed on 12 June 2023) [20]). 

The parameters for the model were chosen as described in the next section, 3.6 Hyperparameters Tuning. Each model underwent 5000 improvement iterations; however, an early stopping criterion of 50 iterations was implemented to avoid overfitting. Scripts of the R implementation can be found in the Supplementary Materials. 

### 3.6. Hyperparameters Tuning

To obtain the optimal hyperparameters for the LightGBM model, we employed Bayesian optimization using the R package ‘rBayesianOptimization’ (https://github.com/yanyachen/rBayesianOptimization (accessed on 12 June 2023) [54]). Bayesian optimization is a method used to find the best solution in a search space by creating a simplified model, often called a surrogate model, to estimate the unknown target function. This approach balances the exploration of new areas in the search space and the exploitation of areas where the model already has some knowledge.

We began by defining the search space for each hyperparameter, considering their respective ranges and the potential for overfitting:

num_leaves: integer values from 8 to 31
max_depth: integer values from 1 to 10
learning_rate: continuous values from 0.001 to 0.9
scale_pos_weight: integer values from 1 to 50
min_data_in_leaf: integer values from 5 to 90
feature_fraction: continuous values from 0.1 to 1
bagging_freq: continuous values from 0.1 to 1
pos_bagging_fraction: continuous values from 0.1 to 0.9
neg_bagging_fraciton: continuous values from 0.1 to 0.9

Where num_leaves controls the complexity of the tree structure, max_depth limits the depth of the tree to avoid overfitting, learning_rate controls the step size during training to balance between convergence speed and optimization performance, scale_pos_weight adjusts the balance between positive and negative class weights, min_data_in_leaf sets the minimum number of data samples in a leaf node to control overfitting, feature_fraction controls the percentage of features used in each iteration to reduce the correlation among trees, bagging_freq controls the frequency of bagging, and pos_bagging_fraction and neg_bagging_fraciton set the fraction of positive and negative bags to use in each iteration for imbalanced data. All the possible parameters can be found in ‘Parameters—LightGBM 3.3.2 documentation’ [55]. As evaluation metric we used the F1-value because it considers the highly imbalanced dataset that we have. The formula can be written as:

(1) $$F1-score=\frac{2\times Sensitivity\times Precision}{Sensitivity+Precision}$$

The dataset utilized for all the hyperparameter optimizations was the WNV dataset. 

We aimed to identify the smallest groups of better-performing peptides and training set, while maintaining a high F1-score. Therefore, hyperparameters were tuned for each of the possible groups to find the best results. Combinations of better performing peptide groups (1% to 40%) and training peptide groups (1% to 10%) were used for the hyperparameter optimization. Detailed R scripts can be found in the Supplementary Materials.

### 3.7. Metric Calculation

In this study, we used the performance metrics, AUC-ROC, accuracy, F1 score, sensitivity, and specificity. The analysis was conducted using R programming language, with packages including ‘caret’ [56], and ‘pROC’ [57].

The best model hyperparameters were obtained as described in the ‘Hyperparameter Tuning’ step from this section and were used to train the LightGBM algorithm. The datasets were treated as described in the section ‘Datasets and Feature Extraction.’ Next, the model was trained and evaluated 100 times through a Monte-Carlo random sub-sampling cross-validation for each data sequence. The model performance was evaluated by generating a confusion matrix using the ‘caret’ package which calculates the metrics of interest, accuracy, sensitivity, specificity, precision (or positive predictive value), and F1 score. The metrics can be defined as follows:

- Accuracy:

(2) $$Accuracy=\frac{TP+TN}{TP+TN+FP+FN},$$

- Sensitivity (TPR):

(3) $$Sensitivity=\frac{TP}{TP+FN},$$

- Specificity:

(4) $$Specificity=\frac{TN}{TN+FP},$$

- Precision (PPV):

(5) $$PPV=\frac{TP}{TP+FP},$$

where *TP* = true positives; *TN* = true negatives; *FP* = false positives; and *FN* = false negative. F1-score was defined in the previous section.

Additionally, the R package ‘pROC’ was used to calculate the AUC-ROC. The ROC curve plots the sensitivity, also known as true positive rate (TPR), against the complement of specificity (1—specificity), or false positive rate (FPR). The AUC-ROC value measures the ability of the classifier to distinguish the positive and negative values.

### 3.8. Data Analysis and Availability

In this study, we conducted data analysis using the R programming language. The packages used for the analysis are: ‘dplyr’ [58], ‘data.table’ [59], and ‘ggplot2’ [60].

The datasets and scripts used in this research can be found in the GitHub repository: ‘https://github.com/jrcodina/LightGBM_Machine_Learning_Peptide_Screening.git’ The repository contains all the necessary data files and scripts to reproduce the results reported in this paper. By providing open access to the data and code, we aim to ensure transparency, and reproducibility.

## 4. Conclusions

In conclusion, our study presents a novel pipeline that significantly accelerates the process of screening an entire peptide space. It has been demonstrated that this method can drastically reduce the time that it takes for such docking screening method, by a factor of at least 10-fold, depending on the chosen strategy. A key distinction from traditional molecular docking is that our method does not score individual peptides. Instead, it utilizes a selection process that categorizes them into two groups in the context of molecular docking ranking: better performers and worst performers. Following this categorization, only the peptides anticipated to be ranked higher are subjected to individual scoring through molecular docking. This approach facilitates a more efficient and targeted screening process, saving time by focusing on promising candidates. Moreover, the structural information about the binding of the selected peptides is not lost.

It is crucial to highlight that this is not a substitute for molecular docking; it rather enhances the screening by shortening the process. Specifically, the proposed process begins with the docking of 1% of the entire peptide space, followed by the application of machine learning. The last step involves using molecular docking for a second time to validate the peptides that show the greatest probability of being suitable as better ligands.

A potential alternative to the initial docking could involve utilizing publicly accessible data; however, the efficacy of this alternative within the pipeline has not yet been evaluated and verified, and it could limit the process to only published data. The proposed pipeline, on the other hand, offers a broad application across virtually any desired target, regardless of whether it belongs into published data domains or not. 

With the growing trend of enhanced computational power, we anticipate that exploring larger peptide spaces will become increasingly feasible and yield a wealth of valuable data. As a natural progression, our future research will aim to scale this process to accommodate larger peptide spaces, like pentapeptides and hexapeptides. We envision that the potential impact of this pipeline could transform the speed and efficiency of bioactive peptide screening, offering new avenues for exploring biological systems and accelerating the development of effective diagnostics and therapeutics where these peptides could be used as binders or therapeutic agents.

## Figures and Tables

**Table 1:** Comparison of various ML methods in terms of processing time and F1-score, obtained from processing the dataset for WNV. The metrics were calculated using a probability threshold of 0.5. The time taken is measured in minutes. The methods include Light Gradient Boosting Machine (LightGBM), Naive Bayes, Recursive Partitioning and Regression Trees (RPART), Gradient Boosting Machine (GBM), Neural Network (NNET), K-Nearest Neighbors (KNN), Random Forest (RF), and Support Vector Machine (SVM).

Method	Time (min)	F1-Score	Accuracy
LightGBM	0.057	0.52	0.85
Naive Bayes	0.874	0.56	0.77
RPART	2.31	0.54	0.84
GBM	20.8	0.56	0.86
NNET	27.7	0.52	0.85
KNN	311	0.55	0.84
RF	326	0.52	0.83
SVM	1690	0.53	0.86

**Table 2:** F1-score across different groups. A Bayesian hyperparameter optimization was performed using a fast search with 15 initial points and 15 iteration points to explore the performance of different group sizes.

“Better Performers” Size (%)	Training Size (%)	F1-Score
1%	1%	0.10
1%	5%	0.13
1%	10%	0.16
10%	1%	0.46
10%	5%	0.43
10%	10%	0.44
20%	1%	0.58
20%	5%	0.58
20%	10%	0.61
30%	1%	0.67
30%	5%	0.68
30%	10%	0.67
40%	1%	0.74
40%	5%	0.75
40%	10%	0.74

**Table 3:** Performance metrics across all datasets, at a 0.5 classification threshold. Groups sizes are 20% for ‘better performers’ group, and 1% for training set group. Optimal hyperparameters were used. For full data, see Table S2. AD = AutoDockFR.

**Metric**	**CHIKV**		**DENV**		**WNV**		**ZIKV**	
	**X**	**σ**	**X**	**σ**	**X**	**σ**	**X**	**σ**
Accuracy	0.85	0.01	0.83	0.01	0.82	0.01	0.85	0.01
Sensitivity	0.76	0.02	0.66	0.02	0.67	0.03	0.73	0.08
Specificity	0.87	0.01	0.87	0.01	0.86	0.02	0.88	0.02
F1-score	0.67	0.01	0.61	0.004	0.61	0.003	0.66	0.07
**Metric**	**CHIKV (AD)**		**DENV (AD)**		**WNV (AD)**		**ZIKV (AD)**	
	**X**	**σ**	**X**	**σ**	**X**	**σ**	**X**	**σ**
Accuracy	0.81	0.01	0.82	0.01	0.83	0.01	0.84	0.01
Sensitivity	0.64	0.03	0.64	0.03	0.66	0.03	0.72	0.03
Specificity	0.86	0.02	0.86	0.02	0.87	0.01	0.87	0.01
F1-score	0.58	0.004	0.58	0.004	0.60	0.004	0.65	0.004

**Table 4:** Five most important features for each dataset from both docking experiments.

OpenEye			AutoDockFR	
Dataset	Feature	Gain	Feature	Gain
CHIKV	Molecular Weight	27%	ProtFP2	29%
	VHSE	26%	T-scales	28%
	ProtFP2	10%	Molecular Weight	5%
	Kidera Factors	10%	Z-scales	5%
	T-scales	2%	Hydrophobicity (Wolfenden)	5%
DENV	ProtFP2	28%	T-scales	53%
	Cruciani (3)	14%	Cruciani (1)	8%
	Molecular Weight	10%	VHSE	6%
	ProtFP3	6%	Fasgai Vectors (6)	4%
	Cruciani (1)	4%	Kidera Factors	3%
WNV	Molecular Weight	45%	T-scales	36%
	PP3	10%	VHSE	13%
	Z-scales	8%	ProtFP2	10%
	Fasgai Vectors	7%	Fasgai Vectors (5)	9%
	Kidera Factors	6%	Cruciani (1)	5%
ZIKV	Molecular Weight	60%	T-scales	28%
	Cruciani (3)	7%	Fasgai Vectors (6)	19%
	T-scales	6%	ProtFP2	7%
	VHSE	4%	Fasgai Vectors (5)	6%
	Kidera Factors	2%	Charge (EMBOSS)	5%

**Table 5:** Time reduction and concurrence of selected peptides. A number of peptides with the highest probability to be better performers, as predicted by the classification algorithm, is selected. These peptides are compared to the peptides obtained by the conventional method by concurrence percentage (percentage of peptides selected by the conventional method that are also selected by the proposed method). Time reduction is calculated by dividing the entire tetrapeptide library (160,000) by the number of selected peptides by ML.

Peptides Selected by ML	Concurrence		Time Reduction Factor
	Openeye	AutoDockFR	
50,000	100%	100%	×3.2
32,000	99%	98%	×5
16,000	95%	90%	×10
8000	85%	81%	×20
4000	69%	67%	×40
2000	50%	51%	×80
1000	33%	38%	×160
500	19%	27%	×320

## Data Availability

The datasets used in this research can be found in the GitHub repository: ‘https://github.com/jrcodina/LightGBM_Machine_Learning_Peptide_Screening.git’ The repository contains all the necessary data files and scripts to reproduce the results reported in this paper. By providing open access to the data and code, we aim to ensure transparency, reproducibility, and ease of collaboration within the research community.

## Supplementary Materials

The following supporting information can be downloaded at: https://www.mdpi.com/article/10.3390/ijms241512144/s1.
