# Supplementary figures and images for: Accelerating the Screening of Small Peptide Ligands by Combining Peptide-Protein Docking and Machine Learning
Source: Int J Mol Sci. 2023 Jul 29;24(15):12144. doi: 10.3390/ijms241512144 (PMC10419121; doi:10.3390/ijms241512144)

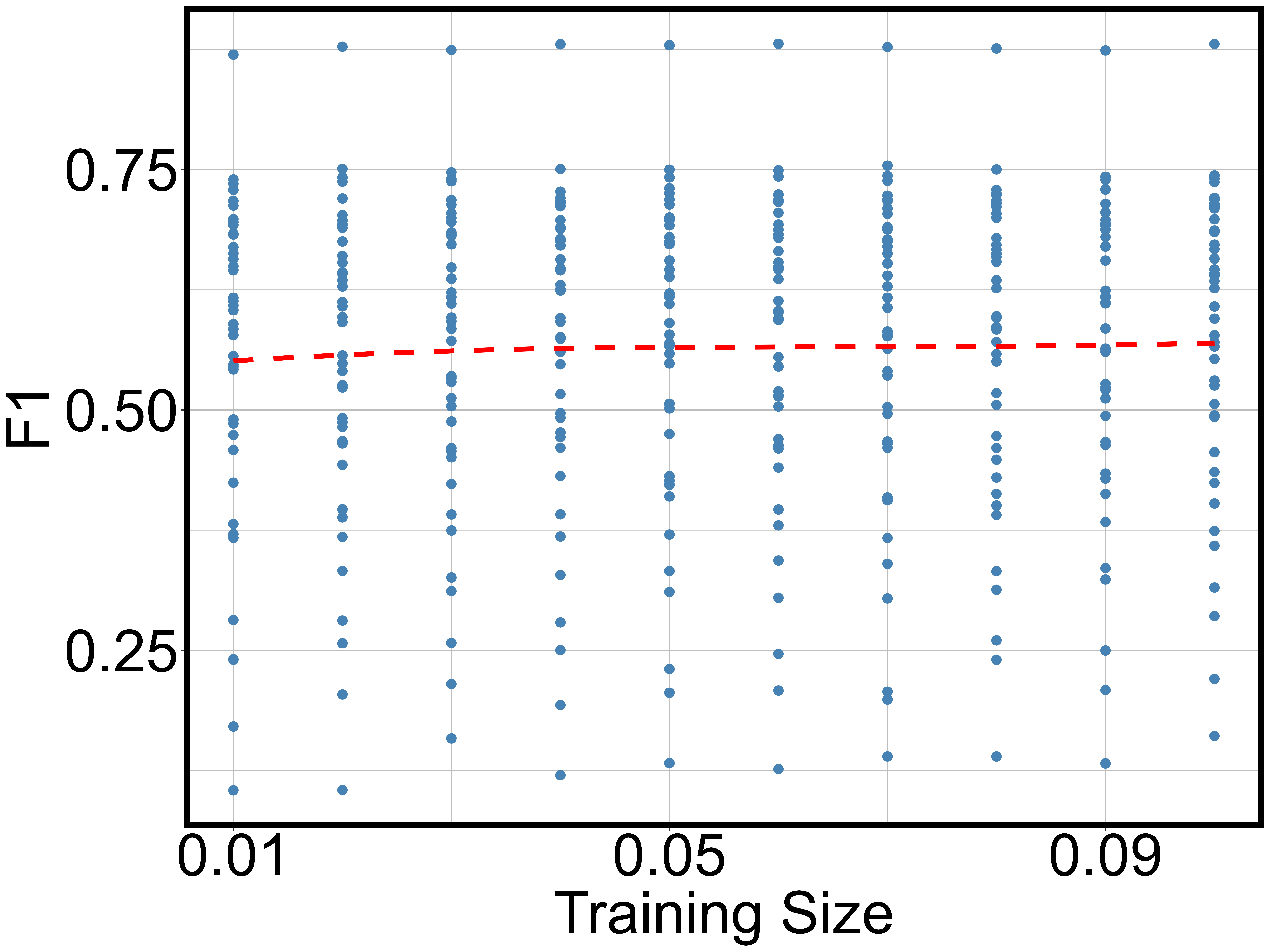

Supplement: Supplementary file 1 [file ijms-24-12144-s001.zip › Figure_S1.a.png]

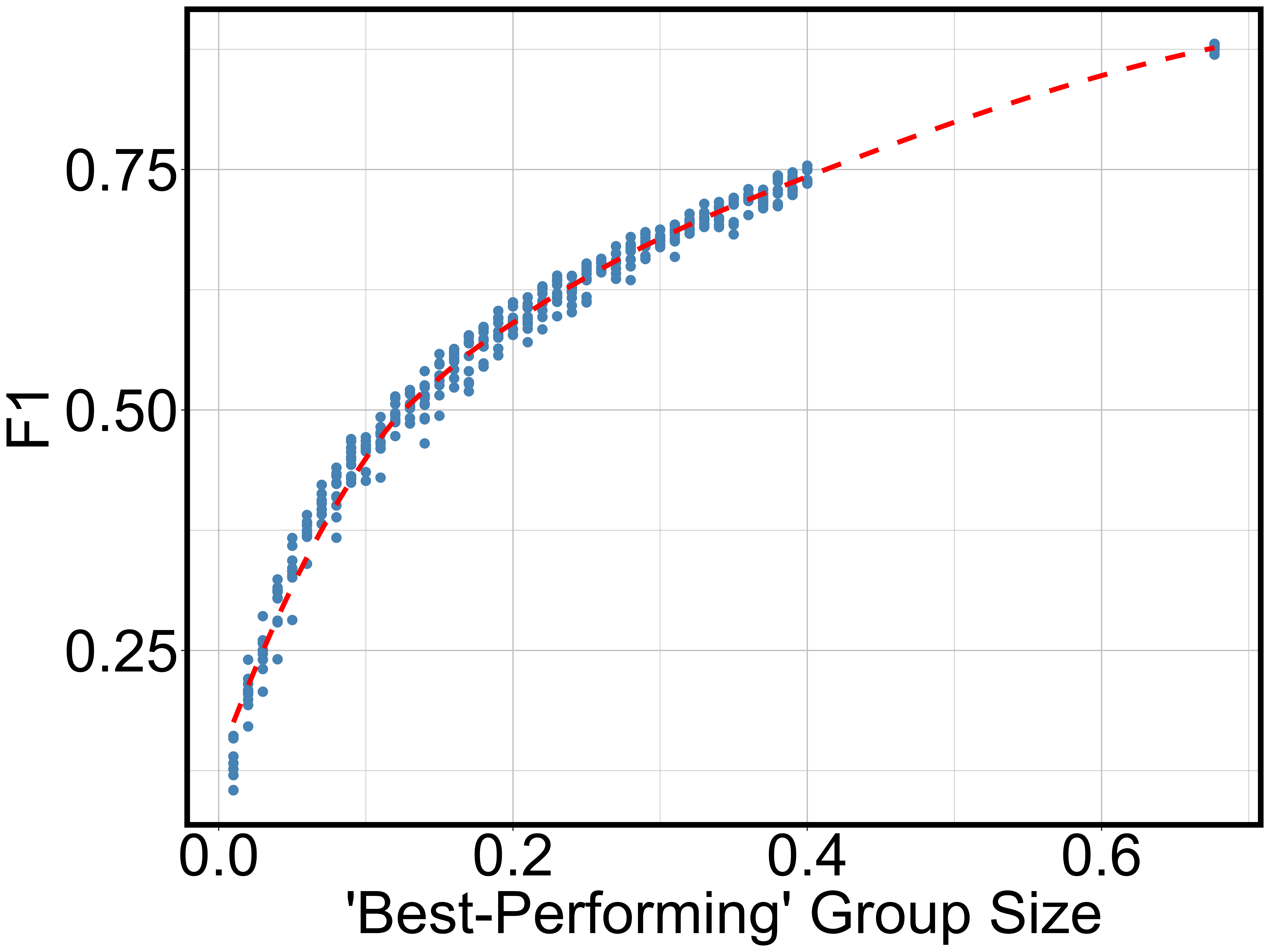

Supplement: Supplementary file 1 [file ijms-24-12144-s001.zip › Figure_S1.b.png]

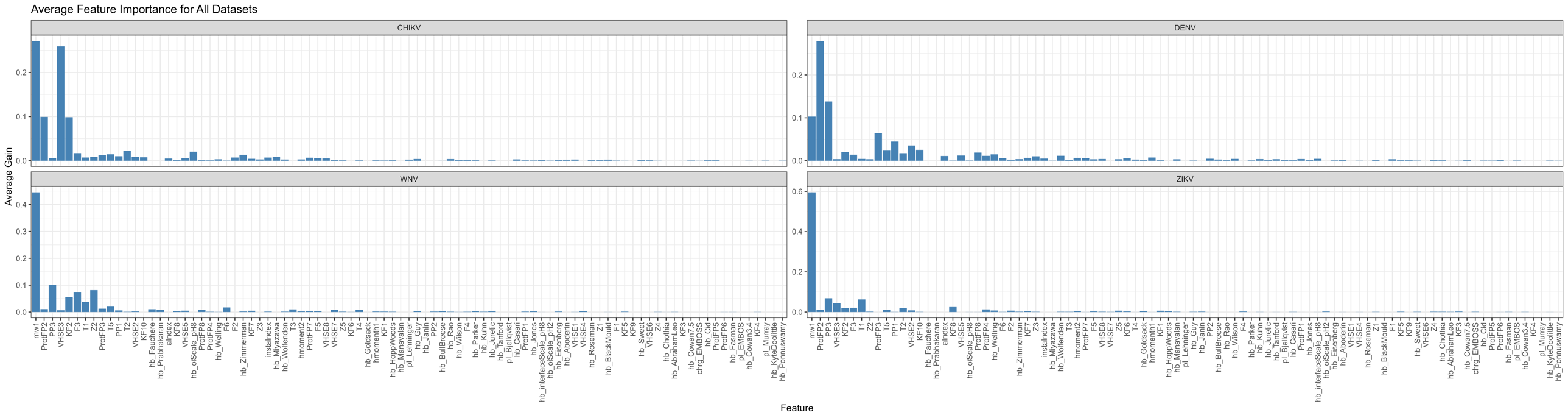

Supplement: Supplementary file 1 [file ijms-24-12144-s001.zip › Figure_S4.a.png]

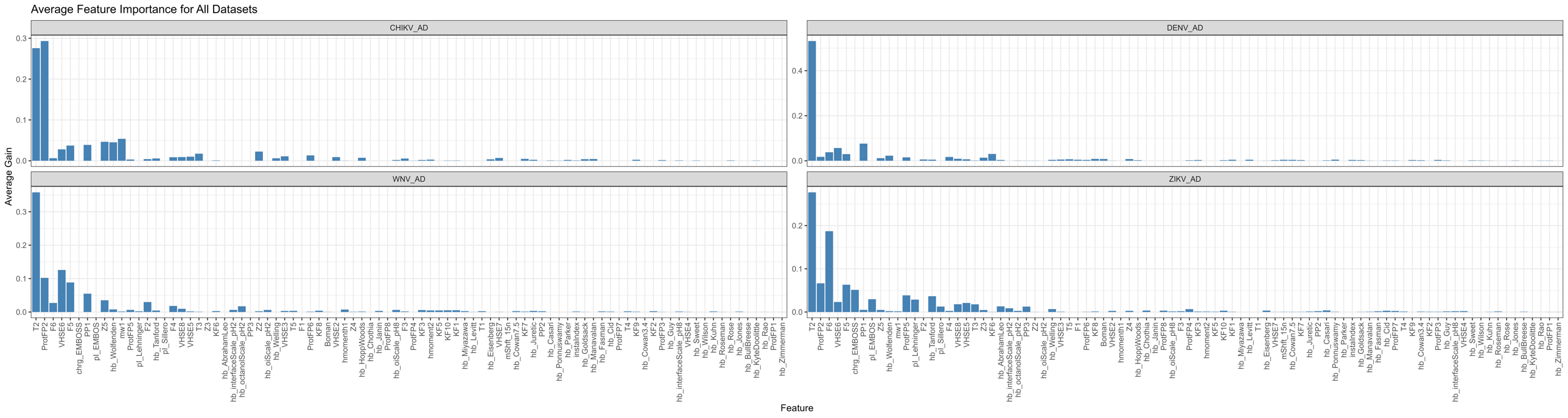

Supplement: Supplementary file 1 [file ijms-24-12144-s001.zip › Figure_S4.b.png]
